# A comparative recognition research on excretory organism in medical applications using artificial neural networks

**DOI:** 10.3389/fbioe.2023.1211143

**Published:** 2023-06-16

**Authors:** Shitharth Selvarajan, Hariprasath Manoharan, Celestine Iwendi, Rakan A. Alsowail, Saravanan Pandiaraj

**Affiliations:** ^1^ Department of Computer Science, Kebri Dehar University, Kebri Dehar, Ethiopia; ^2^ Department of Electronics and Communication Engineering, Panimalar Engineering College, Chennai, India; ^3^ School of Creative Technologies, University of Bolton, Bolton, United Kingdom; ^4^ Computer Skills, Self-Development Skills Department, Deanship of Common First Year, King Saud University, Riyadh, Saudi Arabia

**Keywords:** neural network, digestive systems, loss, attacks, control parameters

## Abstract

**Purpose:** In the contemporary era, a significant number of individuals encounter various health issues, including digestive system ailments, even during their advanced years. The major purpose of this study is based on certain observations that are made in internal digestive systems in order to prevent severe cause that usually occurs in elderly people.

**Approach:** To solve the purpose of the proposed method the proposed system is introduced with advanced features and parametric monitoring system that are based on wireless sensor setups. The parametric monitoring system is integrated with neural network where certain control actions are taken to prevent gastrointestinal activities at reduced data loss.

**Results:** The outcome of the combined process is examined based on four different cases that is designed based on analytical model where control parameters and weight establishments are also determined. As the internal digestive system is monitored the data loss that is present with wireless sensor network must be reduced and proposed approach prevents such data loss with an optimized value of 1.39%.

**Conclusion:** Parametric cases were conducted to evaluate the efficacy of neural networks. The findings indicate a significantly higher effectiveness rate of approximately 68% when compared to the control cases.

## 1 Introduction

The conventional depiction of medical signals does not enable prompt identification of numerous ailments that impact the excretory system of older individuals. The daily routines of elderly individuals are often impacted by inadequate digestion, necessitating the implementation of sophisticated monitoring systems. Therefore, the proposed methodology involves the development of a monitoring system utilizing advanced parametric representation models, with the inclusion of remote monitoring conditions. The majority of systems designed to monitor overall bodily conditions rely on the implementation of sensors to identify any deleterious substances present within the body. The proposed model involves the monitoring of the entire digestive system through the utilization of a low loss device that incorporates distributed weight functions. Furthermore, within the output module, images are acquired and segmented to accurately identify digestive system issues without any hindrance. Enhancement of device security can be achieved through the implementation of control parameters, whereby distinct controlling conditions are specified. It has been noted that many current systems lack control conditions even after the learning stage, as the loss function is optimized for high state conditions. The proposed method involves the integration of a learning algorithm into the monitoring system, resulting in enhanced performance in terms of reduced losses in both the arrangement and analyzer circuit. The learning process involves a comparison of the current and previous states of the digestive system, and if changes are detected after the image segmentation stage, alternative arrangements are implemented.

The cloud monitoring system retains the comparative state of present and past samples, thereby enhancing the security of stored data. During the information storage process, segmented images at a low level are stored. These segmented images are subsequently restored at the output unit, particularly when comparing data history. Consequently, the remote monitoring system saves time by allowing specialists to provide recommendations for individual units at their respective locations. Furthermore, the weight functions are utilized in the hidden layer, resulting in the movement of the device being influenced by high weight conditions. One significant benefit of locomotion in relation to increased body mass is the ability to observe deviations in excretory system function exclusively under specific conditions. Even in low weight conditions abnormality can be detected but serious cause of the problems are defined only if input weights are higher. Furthermore the device arrangement loss can also be prevented by analyzer circuit where elderly people can have low discomfort on wearable device conditions.

**FIGURE 1:**
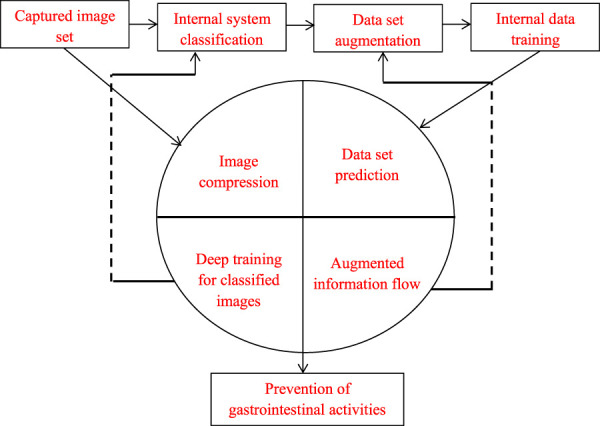
Representation of image data set in digestive monitoring system.

The image set representations that are illustrated in Figure 1 provides a detailed description on data set that plays an important role in digestive system prediction process. The captured image set is completely based on internal system classification that separates the digestive system from other units. Once the data set is separated in to individual unit then data augmentation procedure is carried out where every augmented data is trained using set of reference samples. Once the images are captured entire image is compressed and it is ordered in a way that is clearly visible to senses thereafter every image is classified with deep training neural network algorithm. Further the data flow information that is completely related to augmentation set is checked and arranged accordingly and at last step of process every data is predicted that monitors entire gastro intestinal activities.

### 1.1 Background and related works

The process of identifying digestive issues through remote monitoring systems, subsequent to the input of feeding images, can be elucidated more effectively through the utilization of pre-existing models. These models represent the fundamental components of this detection process. The fundamental concepts of incorporation have been synthesized through the analysis of various researchers’ findings, with a critical examination of established theoretical frameworks. Furthermore, the advancements are carried out with regards to the integration of algorithms as they significantly contribute to the implementation of said procedure. A wearable device has been developed, as described in reference (Zhao et al., 2020), which utilizes image segmentation and sound processing systems to provide physiological indications related to digestive system issues. Despite the incorporation of sound monitoring systems, the frequency of sound is often too low to be perceptible, necessitating the use of a high frequency system in such circumstances. Moreover, this could potentially result in significant harm to the internal auditory canal as a result of the transmission of signals within it. The aforementioned procedure has been transformed into the identification of the small intestine through the utilization of deep learning methodologies (Xiao and Feng, 2020). This involves the implementation of a process for label and feature extraction. The implementation of the labeling technique enables the swift segregation of all images within significantly reduced time intervals. However, if a larger amount of data is present, the time period for detection will increase. Therefore, this method is only suitable for small lacerations. To enable the conversion of the detection procedure for continuous monitoring, a neural network algorithm has been integrated with a set of hidden layers (Wong et al., 2018). This integration has resulted in a high level of control over the transmission of complete data to the intended destination. One significant limitation of this approach is that neural network capabilities are only applicable to closed-loop operations, implying a preference for manual procedures during extended periods of continuous monitoring.

In addition to the aforementioned algorithms, a food intake system analytical model has been developed to facilitate the appropriate monitoring of model parametric values (Chudtong and De Gaetano, 2021). The utilization of mathematical models results in the establishment of an open loop automatic configuration system, wherein each determinant within the process imparts knowledge pertaining to the nature and magnitude of the food. However, it is possible to monitor the quantity of food present in the digestive system as it undergoes breakdown within the body of an individual. From a conceptual standpoint, only a limited quantity of ingested material can be identified through specific components that are present within the gastrointestinal tract. On the other hand, the analytical model is expounded through neural formation networks comprising three discrete compartments, namely, stiffness, nutrition, and medication rate, which must be ingested at appropriate intervals (Guerrero Sánchez et al., 2020). The aforementioned system is beneficial to elderly individuals, as it offers daily information that is automatically stored in a database. The factorial model is utilized to obtain numerical solutions for the three compartments model by deriving differentiable equations.

The primary significance of attaining results through analytical equations is also formulated utilizing the wavelet transformation method (Srinivasa et al., 2021), which was introduced for COVID identification. The present system is designed to assess potential harm inflicted upon the complete digestive system in the presence of an infectious viral agent. This form of detection is particularly beneficial in times of pandemics, as it enables the administration of ongoing treatment to prevent loss of life among individuals. Furthermore, a defensive mechanism for strategy has been formulated through the utilization of various learning models (Lee et al., 2021), which is commonly referred to as the defensive neural network algorithm. Incorporated learning models will be utilized to detect high severity cases, with the aim of minimizing the occurrence of various types of attacks. In addition, it is noteworthy that the data processing capacity of said techniques is endowed with robust security measures, whereby confidential information is stored in local server stations. Subsequently, upon assessing its necessity, the distribution of the aforementioned will be extended to multiple networks, ultimately resulting in heightened efficacy. In order to enhance the amplification of the digestive system, it is necessary to incorporate a greater quantity of fluid dynamics, as indicated by reference (Ferrua and Singh, 2010). The integration of unique insight feature sets is aimed at achieving comprehensive understanding of digestive systems. In addition to the acquisition of knowledge, the results are evaluated in three-dimensional scenarios to observe distinctive characteristics in intricate mechanisms. In order to effectively process three-dimensional images, it is imperative to utilize multiple diagnostic mechanisms through hierarchical procedures, as indicated by reference (Sali et al., 2020). Consequently, the implementation of neural networks in the organization of histopathological images involves the utilization of hierarchical procedures. The images presented offer comprehensive insight into the depth of the digestive system, complete with clearly labeled components, facilitating the identification of affected regions with ease. Upon careful examination, it is evident that the expense associated with acquiring an image set is significantly greater, thus rendering it unfeasible to accurately segment minor anomalies within the gastrointestinal tract.

There exists a significant likelihood that the digestive systems may be impacted as a result of irregularities in the reproductive axis, wherein discerning additional physiological alterations may prove challenging (Zavala et al., 2019). Therefore, in order to regain typical operational capabilities, a fundamental parametric detection approach is employed through the establishment of a mathematical model. The utilization of multiple tools can result in a heightened level of complexity within the analytical model, leading to challenges in achieving desired outcomes. Abnormalities in the digestive system can be detected through a two-state terminology process that incorporates active and passive cell modeling features when slower waves are present (Du et al., 2018). The present study employs tissue models to actively participate in high cell detection forecasting. However, the identification conditions become unstable due to the increased number of identical tissues. The detection, processing, and location of image sets have been the subject of discussion in conjunction with conceptual systems, with various mechanisms introduced for review purposes (Chambers et al., 2014; Du et al., 2019; Gan et al., 2020; Populin et al., 2021). Upon analysis of these fundamental elements, distinct sets of characteristics have been identified for the purpose of constructing a system model for the proposed method.

### 1.2 Research gap and motivation

All the existing methods that provides monitoring state using entropy functions defines only necessary problems that are related to current situation. As a result of such monitoring process there exists low probability of success even if partaking analyser is introduced in the process. However some of the basic developments that leads to gastro intestinal activities and their corresponding attacks are not discussed. It is always necessary that in examination of excretory organism most of the digestive problems are clearly monitored with conditional image set. But the information from the image set is present with unmarked points thus leaving other distinct characteristics to remain at unstable state. Due to absence of such differentiation the existing system contains maximum loss that cannot be prevented and only 30% digestive problems are detected.

In order to solve the above mentioned gap, proposed method is implemented with feature set that contains entropy functions with distributed weights. Moreover the design model is introduced with control parameters therefore the outcomes in the images that identify the excretory organism remains in stable conditions. However with distributed weights partaking analyser is introduced with time series representations therefore according to varying time factor the monitoring periods are considered. Additionally to increase the efficiency in detection of excretory organism neural networks are introduced with discourage loss that remains lower than 5%. As a result of measurement in loss periods the proposed method reduces the monitored attacks by following conditional assignments.

### 1.3 Contributions

The major contribution of the proposed method depends on monitoring the digestive system changes in elderly people where remote data is processed using neural network algorithm and the parametric objectives are provided as follows.

• To measure the entropy functions with relatable mini data type by representing the input data samples with low loss conditions.
• To establish distributed weight functionalities using partaking analyser where equal weights are distributed across distinct signals.
• To minimize the loss in signals and data attacks by following certain conditional assignments thus determining the learning rate of proposed method.

### 1.4 Structure

The rest of the paper is organized as follows: Section 2 provides the system model with relevant analytical framework. Section 3 integrates the system model with neural network algorithm to increase the efficiency in detection process. Section 4 provides outcomes for the designed method with four cases and discussions based on simulation outcomes are added. Finally Section 5 concludes the paper with directions in future work.

## 2 Proposed system model

The issue of indigestion and recurrent episodes can be addressed through various educational methodologies. An analytical model is required to address issues pertaining to the digestive system, whereby the learning parameters can be formulated using the equations presented below. During the initial phase, the learning parameters will be incorporated, and subsequently, after resolving and differentiating the learning parameters, the tuning algorithm will be integrated. Eq. 1 can be utilized to express the entropy function in the following manner (Chudtong and De Gaetano, 2021).

$$A_{i}=\sum_{i=0}^{n}\frac{l_{i}\left(m_{d}\left(i\right)+P_{d}\left(i\right)\right)}{\partial m_{d}\left(i\right)}$$ (1)

Where, $l_{i}$ represents the loss function of input samples

$m_{d}\left(i\right)$ and $P_{d}\left(i\right)$ denotes the mini data and private data set of input images

$\partial m_{d}\left(i\right)$ indicates the differentiable mini data type

The abovementioned Equation will be fed as an input to a digestive device analyzer that trains the input data using average weight parametric values. These weights can also be distributed across all devices without any external disturbance by following corresponding protocols. Thus the distributed weight equation can be represented as,

$${DW}_{i}=\sum_{i=1}^{n}\frac{{wR}_{i}}{N}$$ (2)

Where, ${wR}_{i}$ indicates the distributed weight across networks of digestive system

N denotes maximum weight of the partaking analyzer

Since the weights are distributed across different systems the signals will also vary according to the time series representation using angle metric as follows (Srinivasa et al., 2021),

$$T_{i}=\sum_{i=1}^{n}A^{T}\varphi \left(i,n\right)$$ (3)

Eq. 3 indicates that variations in angle will make the signals to move across different directions thus providing different values for representing time periods. Thus the learning rate of collaborative parameters can be represented using minimization of loss formula using Eq. 4 as follows (Lee et al., 2021),

$$D_{l}\left(i\right)=\min\sum_{i=1}^{n}C_{l}\left(i,n\right)+\mu_{i}$$ (4)

Where, $C_{l}\left(i,n\right)$ indicates the arrangement loss

$\mu_{i}$ represents the discourage loss of analyzer circuit

The control performance of the analyzer circuit determines the point that is set at regular intervals using time step periodic functions. In addition if the analyzer is not having any control parameter then distributed weights in Eq. 2 will not be properly distributed and as a result of it attack strategy will be formed. Therefore control parameter can be framed using Eq. 5 as follows (Ferrua and Singh, 2010),

$$C_{i}=\min\sum_{i=1}^{n}\frac{1-{attack}_{i}}{{SZ}_{i}}*100$$ (5)

Where, ${attack}_{i}$ represents the attacked distributed circuit

${SZ}_{i}$ denotes the size of the circuit

If there is an attack in the circuit then the dataset must be checked properly in order to make the attack free from external systems. This can be represented using Eq. 6 as follows,

$${attack}_{i}=\sum_{i=1}^{n}\left(r_{i},s_{i},t_{i}\right)*\left(r_{n},s_{n},t_{n}\right)$$ (6)

Where, $r_{i},s_{i},t_{i}r_{n},s_{n},t_{n}$ indicates the attacks in *i*th and *n*th digestive analyzer loops

Eq. 6 is subject to certain conditional assignments as follows,

$$r_{i},s_{i},t_{i}r_{n},s_{n},t_{n}=\left\{\begin{array}{c} 1\;if\;i\in n\\ 0\;otherwise \end{array}\right.$$ (7)

Eqs 1–7 represents design if three dimensional device that detects the digestive system of all individuals. However it cannot be incorporated without the integration of neural networks as separate images must be fed as input for identification process. This integration process is represented in Section 3.

## 3 Optimization algorithm

The incorporation of optimization algorithms in the identification of the digestive system is imperative in order to enhance identification efficiency and reduce the costs associated with system design implementation, as noted in sources (Garg, 2019; Mali et al., 2021; Eq. uations, 2022; Shafiq et al., 2023a; Shafiq et al., 2023b; Shafiq et al., 2023c; Çolak et al., 2023). The most effective algorithm utilized for identification purposes is commonly referred to as a neural network. This involves the implementation of a group of neurons within the system, which are simulated under various conditions using iteration values. Furthermore, the present study involves the development of a system of nonlinear equations based on the specified objective functions, utilizing forward propagation matrix parameters as outlined in Eq. 8.

$$P_{NN}\left(i\right)=\sum_{i=1}^{n}\sigma_{in}\left(\theta_{i}+w_{i}\right)$$ (8)

Where, $\sigma_{in}$ indicates the sigmoid function of input and hidden layers with data information loop

$\theta_{i}$ and $w_{i}$ represents the hidden layer parameters

The parametric values in Eq. 8 will convert the output values to optimum thus achieving gradient elements calculating total error functions in the output process. Therefore the parametric determinations of the output units can be represented using Eq. 9 as follows,

$$E_{i}=\min\sum_{i=1}^{n}\frac{\partial \theta_{i}}{\partial_{i}}-L_{i}$$ (9)

Where, $L_{i}$ represents the learning rate of differentiable parameters

In Eq. 9 the gradient function is added for entire parameters but in real time implementation it must be added separately which is designed using Eq. 10 as follows,

$${cost}_{i}=\min\sum_{i=1}^{n}\left(w_{i}^{2}-\theta_{i}^{2}\right)$$ (10)

In Eq. 10 the difference between weight of installation and number of parameters in hidden layers will determine the cost function. However the usage of back propagation matrix can also be determined for minimizing error functions using input and hidden layers as follows,

$$E_{i}\left(backpropagation\right)=\min\sum_{i=1}^{n}\frac{w_{i}}{w_{i}+w_{n}}*E_{in}$$ (11)

From Eq. 11 the total error function can be calculated by switching the rows and columns of consecutive matrix type. Therefore the output value can be represented using Eq. 12 as follows,

$${out}_{i}=\left\{\begin{array}{c} 1\;if\;w_{i},L_{i}>\theta_{i}\\ 0\;otherwise \end{array}\right.$$ (12)

Eq. 12 indicates that abnormal units in digestion system will be indicated only of conditions are satisfied. Otherwise the system will produce a constant zero representation. Therefore if any loss values are identified then it can be determined using Eq. 13 as follows,

$${loss}_{i}=\frac{1}{2}\sum_{i=1}^{n}\theta_{i}-\hat{\theta_{i}^{2}}$$ (13)

The difference between original and reference values provides exact loss functionalities but high average prediction can be achieved only of sum terms are integrated. Therefore Eq. 13 can be modified as follows,

$${modified\;loss}_{i}=\frac{2}{n}*sum\left({loss}_{i}\right)$$ (14)

**FIGURE 2:**
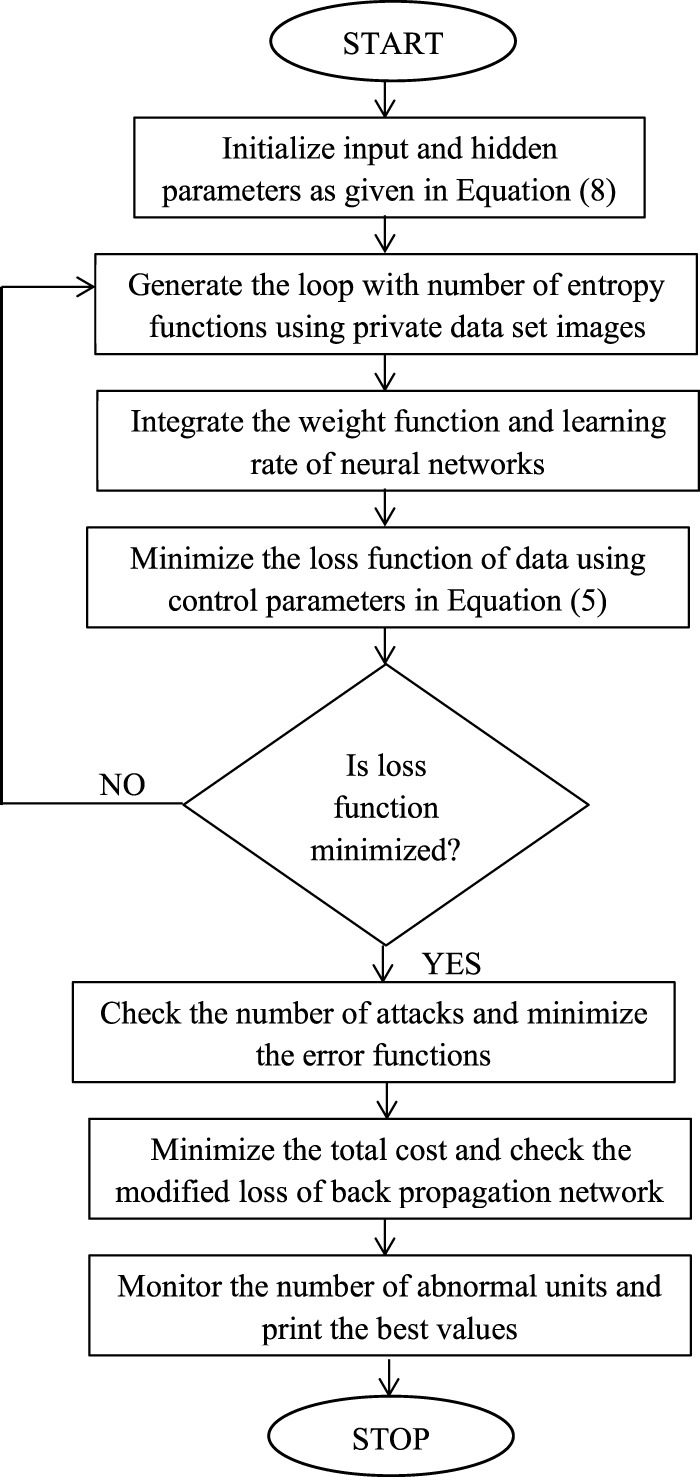
Integration of neural network with system model.

Table 1 provides hyper parameters of back propagation neural network and the algorithmic flow chart in Figure 2 specifies the step implementation of analytical equations in simulation segments whereas in real time setup these steps are not needed and a direct setup using a set of neural signal can be built for observing the output values.

**TABLE 1:** Hyper parameters of back propagation neural network.

Hyper parameters	Values
Non-linearity	0.5
Learning rate	2.4
Impetus	0.9
Number of epoch	10 to 100
Image dimension	32 × 256
Number of hidden units	125
Number of input units	212

The design model in the proposed method is generated based on back propagation neural network where initial weights are tuned for reducing the error rates. In addition, clear information about design model for detecting neural digestive problems can only be achieved if inputs and hidden weights are minimized as high data processing is needed in the detection problem (Khadidos et al., 2023; Kshirsagar et al., 2022; S et al., 2022; Shitharth et al., 2022). However it is essential to check the hidden units as the designed device can able to provide additional data weights inside the monitored area where gastro intestinal activity occurs. Hence the design model consists of forward and backward pass measurements that are calculated using weights and biases thereby total error functions with addition of hidden and input units are determined. In other way the design model is also based on entropy functions that detect exact probability values in the connected network. Once the design model output is summarized then activation functions are determined using new weight and bias connections. If the design model is appropriate then the incoming signals will be directed to layers 1 and 2 therefore every output function is based on reference values in the data set and values that are present with previous samples.

The flow chart that is presented in Figure 2 represents the integration steps that are used for detecting entire digestive system for elderly people where data transmission efficiency is maximized with low loss. In the first step every designed parameters are initialized in both input and hidden layers where a sigmoid function is achieved. By using the sigmoid function entire image set of inner digestive system is captured and entropy functions are defined by examining the amount of loss samples in captured images.

If the loss functions are much higher then distributed weight functions are defined according to partaking analyser thereby the learning rate of output units are achieved. Further if the data set is accurate with maximization values then it is possible to minimize the loss functions thereby the gastrointestinal attacks are prevented by examining the output image set. In case of abnormal unit detection separate images are generated and it is transmitted to control unit thereafter modified loss functions are determined. In the proposed method entire data set is trained only for comparing the reference values that is carried out using back propagation neural networks. During the process of training, external errors are added in the system therefore every measurement values in rows and columns are shifted by establishing consecutive type system. In addition the data set that needs to be trained is not tuned with any parametric representations therefore no prior knowledge on existing data set is needed for computing the current output values and monitoring states. The major intention of choosing back propagation neural network is that there is no need of establishing any special function to defined data set therefore original characteristics of monitored image output can be recovered.

## 4 Results and discussions

This section presents a description of the effectiveness of the analytical model and neural network algorithm in real-time testing cases through their combined outcomes. It should be noted that the integration of healthcare-related components necessitates extensive testing, hence the proposed method only presents simulated models. In addition, the hardware configuration facilitates the collection of ongoing monitoring data that is transmitted directly to MATLAB in real-time mode. This configuration facilitates a lucid comprehension of integration scenarios, as the software application is primed for efficient operational functionality. During the training phase, a set of 200 images depicting the digestive system are captured for each individual, and subsequently utilized for training purposes. Upon automatic installation of input images into the system, segmentation of digestive images is performed at various points to precisely identify any underlying issues. Furthermore, in conjunction with the aforementioned phases and conventional parametric representation, the testing phase is conducted across four distinct scenarios, outlined below.

Case 1: Formation of entropy functions

Case 2: Distribution of equal weights

Case 3: Minimization of modified loss function

Case 4: Identification of control parameters and attacks

All four cases provide correct parametric values for testing images, thus indicating that no usual simulation cases are followed. Further the abovementioned case studies are simulated using a loop formation matrix which is much essential for image segmentation and classification process.

### 4.1 Case 1

The entropy function is commonly utilized to detect stochastic irregularities in particular functions, and it is derived from axon segments of neural networks. The methodology involves partitioning the complete dataset into smaller subsets to facilitate precise observations and expedite the testing timeline. Furthermore, a confidential dataset that requires comparison with a smaller dataset will be preloaded during both the training and testing stages. Consequently, prior to conducting original image testing, the training images of confidential data will undergo processing and will be designated as a loss function within the system. Entropy is a significant parameter for monitoring the gastrointestinal segment due to its representation of biological functions and suitability as a transmission segment. The detection of the path of heat flow within a component system can be facilitated through the determination of the entropy function. Hence, in the event that the temperature decreases significantly, the mini data will undergo differentiation, resulting in the formation of novel channel matrix values that are appropriate for transmission.

**FIGURE 3:**
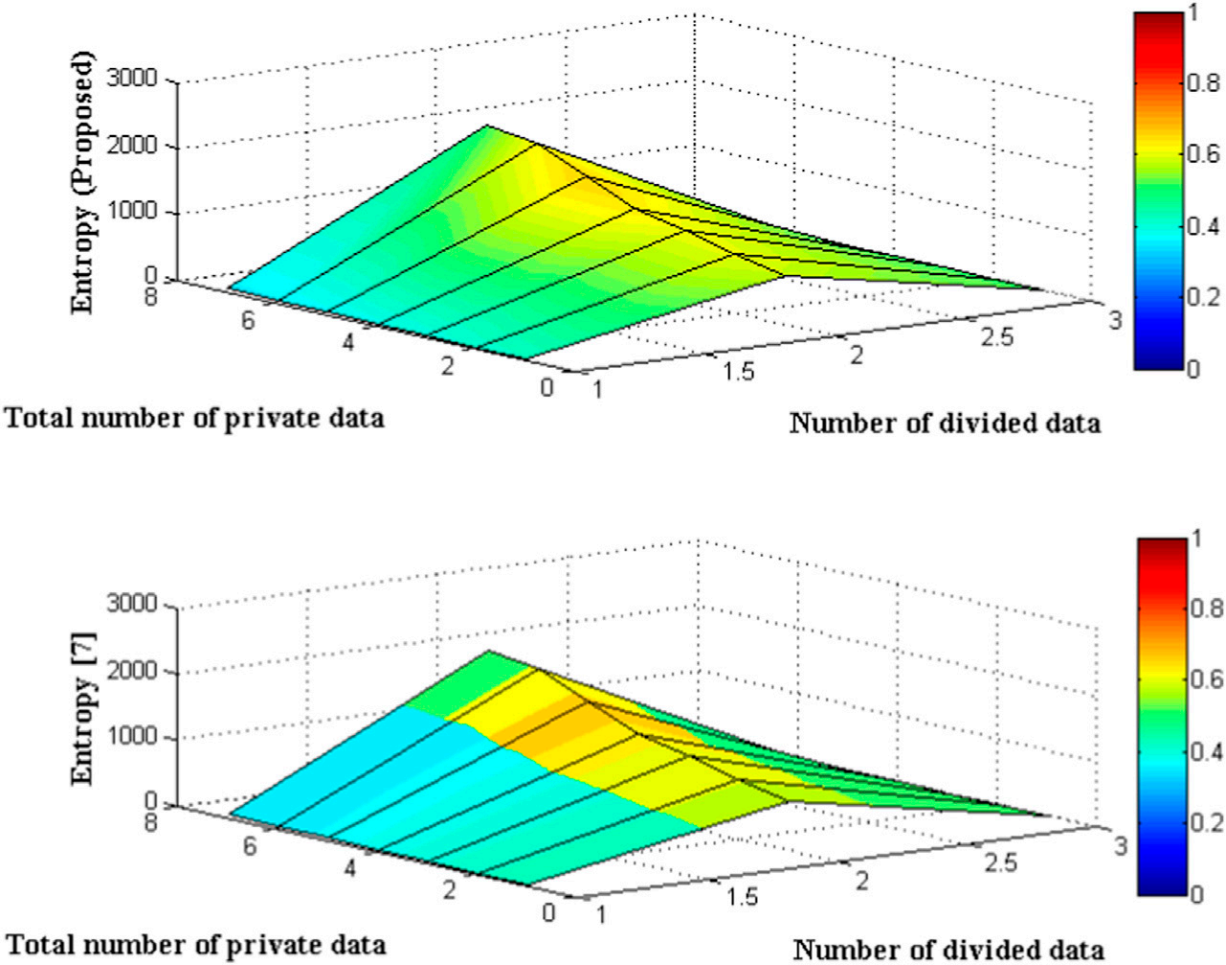
Comparison of entropy functions with divided data set.

The entropy function, calculated using a mini and private data set, is presented in Figure 3 and Table 2. The input values for this function consist of seven distinct private data points. The complete private dataset has been partitioned into subsets of sizes 8, 10, 14, 19, 24, 39, and 45, which are being utilized for the purpose of processing the input image set. Thus, these segregated sets will operate for a brief duration, and upon completion of the entire process, the data values will be stored within the system. The storage of values can be facilitated through an authenticated cloud system. However, as a result of comparison, the entirety of the data is stored within an integrated tool. Subsequently, the computation of entropy is conducted, whereby the observed quantities are required to fall within the threshold of 1. However, the current approach overburdens this circumstance as a result of inadequate partitioning of confidential information, thereby surpassing the threshold of unity. On the other hand, the method suggested utilizing neural network remains within the established threshold and once again attains appropriate saturation levels upon completion of the entire dataset.

**TABLE 2:** Entropy with segmented set of data.

Number of divided data	Total number of private data	Entropy Lee et al., (2021)	Entropy Zavala et al., (2019)	Entropy (proposed)
8	744	0.4	0.6	0.1
10	894	0.8	0.9	0.3
14	1,088	1	1.3	0.7
19	1,264	1.1	1.4	1
24	1,580	1.1	1.5	0.5
39	1897	1.2	1.5	0.2
45	2014	1	1.1	0.1

### 4.2 Case 2

The successful operation of a neural network in image detection necessitates the utilization of weight matrix functions that are uniform in nature. This is crucial in preventing the manifestation of segment errors during the simulation process. A specialized device, known as a participatory analyzer, has been developed for the purpose of detecting the maximum weight of an input matrix. The primary rationale for investigating the weight function pertains to the potential variation in digestive system function that may occur in the presence of a significantly elevated body mass index in an individual. Unequal distribution of weights can result in unclear information from images, necessitating extensive testing that can be a time-intensive undertaking. Hence, it is imperative to ensure equilibrium in the weights of input neurons under both static and dynamic circumstances, in order to prevent significant modifications prior to their transmission to the hidden layers. The distribution of weights carries a significant level of risk. However, the digestive system’s five-layer process of neurons ensures efficient processing.

**FIGURE 4:**
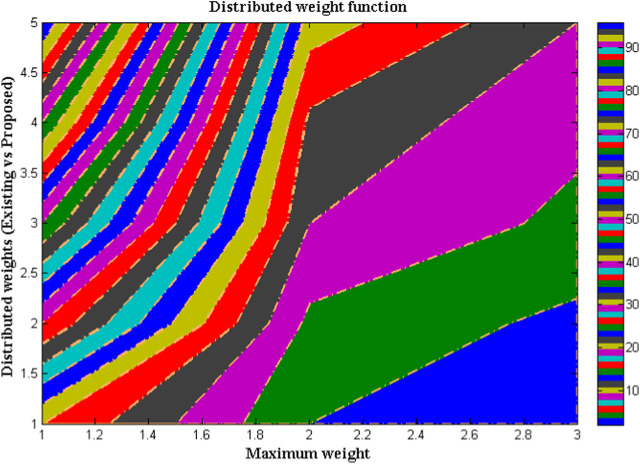
Comparison of distributed weights.

The comparison of equal distribution between the existing method (Lee et al., 2021) and the proposed approach is illustrated in Figure 4 and Table 3. In order to facilitate a comprehensive comparison, the total number of weights, denoted as N, has been partitioned into five segments: 20, 40, 60, 80, and 100. Within each of these segments, an equitable distribution of weights has been allocated. It is widely acknowledged that weight distribution should not exceed 12% of the total allocated weight. However, conventional methods typically permit a maximum of only 10%. The proposed method is capable of achieving a uniform distribution of weights, even with standard values, in contrast to the existing method during the initial stage when the input images are of lower resolution. Upon increasing the image segmentations, the proposed method attains an equitable allocation of weights for approximately 11%, thereby implying a reduction in the potential hazards associated with the installation and processing of image segments. Moreover, the proposed method satisfies the condition even at its maximum weight, and can be applied to other large input data fields.

**TABLE 3:** Distribution of maximized weight functions.

Maximum weight	Distributed weights Lee et al., (2021)	Distributed weights Zavala et al., (2019)	Distributed weights (proposed)
20	4	6	2
40	7	7	3
60	12	11	7
80	15	16	9
100	22	23	12

### 4.3 Case 3

The weight updating process is evaluated through loss functions, which involve the use of summation terms in the measurement. The method of introducing certain terms is commonly referred to as modified loss functions, which are utilized for the computation of gradients for each function. The loss function evaluated for neural networks exhibits variations between forward and backpropagation networks. Specifically, high loss functionalities of a given image are manifested at the input stage. However, in the output stage, the reduction of image loss during classification is observed. This method of minimizing the loss function identifies the likelihood of hazardous content in gastrointestinal processes. The proposed method requires the use of a tensor flow in order to predict the stages of the neural network, thereby providing precise measurements of cross entropy functions in scenario 1. Consequently, the present methodology involves the organization of data utilizing neural networks in a sequential manner, with the optimal parameters being ascertained accordingly. Furthermore, this particular loss function establishes a direct correlation between the input and output layers of a neural network.

**FIGURE 5:**
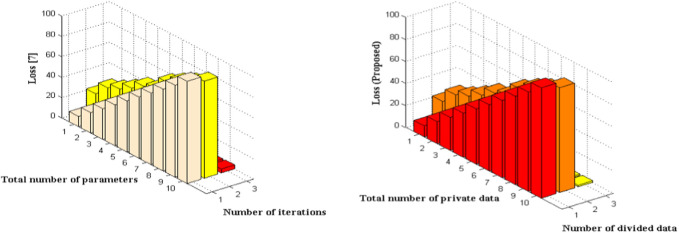
Comparison of loss in neural network.

The minimized loss functionalities of the proposed method and a comparison of loss with an existing approach (Lee et al., 2021) are indicated in Figure 5 and Table 4. The system incorporates a hidden layer parameter derived from the reference value of the dataset. The modification of values is processed by the system, and subsequently, these altered values are transmitted to the sum functionalities through a specified number of iteration periods or time segments. Therefore, a prolonged sequence of 100 iterations is analyzed with a maximum parameter count of 95 per division. By utilizing these standard specifications, the simulation results were evaluated and it was observed that the proposed approach employing neural networks effectively decreases the modified loss to 1.39 decibels. The existing method experiences a significant increase in loss as the number of iterations is increased, resulting in a high value of 3.76 decibels.

**TABLE 4:** Total parametric loss of network.

Number of iterations	Number of parameters	Loss Lee et al., (2021)	Loss Lee et al., (2021)	Loss (proposed)
10	27	1.86	1.62	0.47
20	39	1.94	1.79	0.49
30	44	2.08	2	0.57
40	51	2.19	2.06	0.83
50	59	2.2	2.12	0.95
60	63	2.36	2.24	1
70	75	2.81	2.27	1.18
80	82	3.27	2.48	1.23
90	88	3.52	2.94	1.29
100	95	3.76	3.05	1.39

### 4.4 Case 4

Parametric analysis is a crucial aspect of circuit design for gastrointestinal activities, as it involves identifying the requisite control parameters. The determination of circuit size is commonly regarded as reliant on the utilization of a distributed data set throughout the entire process. Furthermore, conditional assignments for data transfer stages are established as control parameters to prevent system attacks. Although the two relationships are mutually exclusive, the quantity of loops in the system is determined by control parameters, thereby integrating the process of attack determination. The primary reason for investigating this case is that neural networks possess an inherent capacity to autonomously determine the model and adjust to specific contexts. However, this approach may not be suitable for identifying potential hazards in gastrointestinal functions, as even minor alterations in any given variable can result in significant harm to an individual. In addition to the aforementioned significance, neural networks have been acknowledged as a more suitable option for parametric assessment in comparison to alternative algorithms.

**FIGURE 6:**
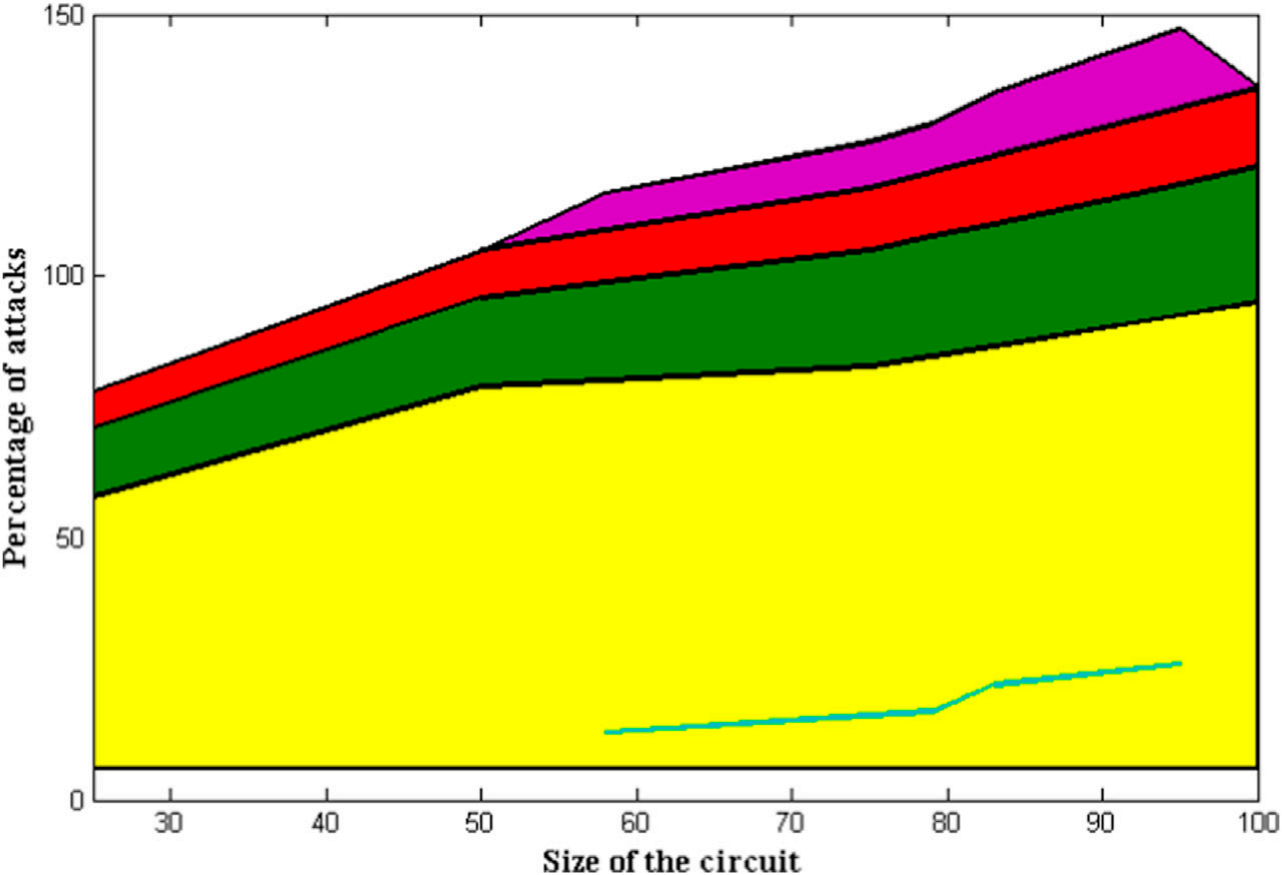
Attack with control parameters.

The simulated pageant of control parameters is specified in Figure 6 and Table 5, utilizing the size of the device and percentage of attacks. It is evident that a limited number of control parameters are required for the neural network to effectively train and test the data, as indicated by the comparison. In contrast, the present approach necessitates fewer parameters for the same configuration, with a maximum of 26 parameters required during significant attack intervals, as compared to the previous method (Lee et al., 2021). The present study showcases the efficacy of the proposed technique in addressing the issue at hand. Specifically, when the device size is set at 100 and the percentage of attacks is at 95, the method in question necessitates a mere 15 control parameters to effectuate the conversion of the process to a state of normal functionality. The decrease in control parameters is anticipated to result in a reduction in installation expenses and a decrease in the number of black boxes that are incorporated into the system as input functions.

**TABLE 5:** Comparison of control parameters.

Size of the circuit	Percentage of attacks	Control parameters Lee et al., (2021)	Control parameters Lee et al., (2021)	Control parameters (proposed)
25	58	13	11	7
50	79	17	14	9
75	83	22	18	12
100	95	26	22	15

### 4.5 Uncertainty analysis

**FIGURE 7:**
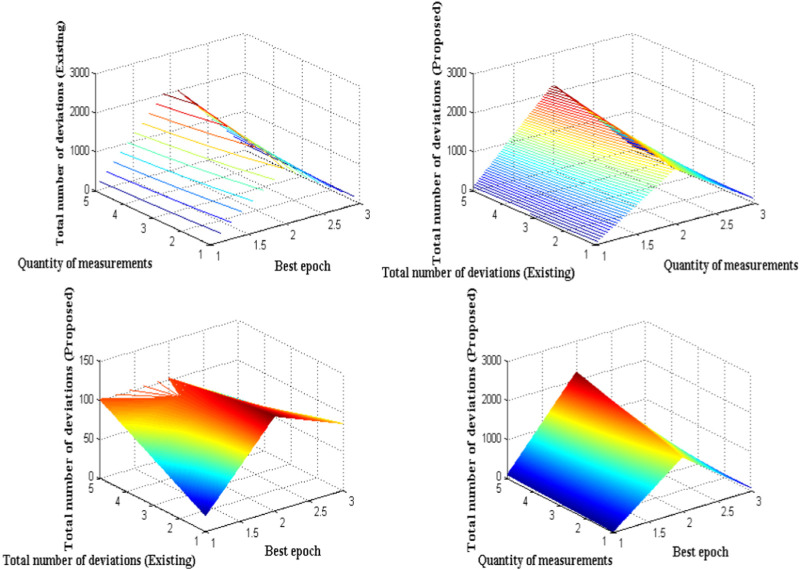
Uncertainty analysis in terms of deviation measurements.

Even though the proposed method provides a unique solution for monitoring entire gastro intestinal activity some problem occurs due to improper decision during data transmission process. It is observed that at monitoring stage appropriate data is present whereas during data transfer state as two set of images are taken by considering mini and private data. Thus some of the data results in improbability analysis that is analyzed in this case study. In the process of monitoring and data transfer the standard measurements are taken by considering absolute values where input quantity is directly related with some of the uncertainties that are represented in measurement model. Moreover there is a possibility that device measurements results in uncertain state by following inconsistent values thereby the quantity of measurements results at undefined state of operation that needs to be avoided. Further the deviated value from original reference value must be compared for converting the uncertain monitoring cases to normal operational cases. Figure 7 provides uncertainty measurements with deviated values.

The total number of deviations in Figure 7 indicates that uncertainty is minimized for proposed method as compared to existing approach (Lee et al., 2021). To verify uncertainty case in simulation five best epoch values are considered from 20 to 100 where the quantity of measurements changes from low to high once the state changes are increased. Therefore the lower and upper limits are considered as 1,400, 1,600, 1,800, 2,000, and 2,200 respectively where fore every quantity measurements total number of deviations is measured and it remains at 121, 117, 113, 107, and 101 whereas for proposed method deviation values remains at 85, 76, 63, 54 ,and 48 respectively. It is also further possible to control more amount of deviation in the data transmission process by reducing total loss in measurement process. The proposed model on neural digestion with back propagation neural network is implemented with huge data set where deviations are identified. In all the indicated data set it is observed that most of the data samples are identical to original values where 32 × 256 array is introduced. Hence the deviation from original value must be identified in order to monitor entire gastro intestinal activities that are present in an individual. The deviation can only be realized if the distributed weight functions are represented with partaking analyzer. Further with weight deviation it is essential to vary the time representations of every signal thus maximum accuracy is achieved. In case if time period representations are not varied then accuracy of monitoring remains constant and no variations are considered. If no variations are found then loss in data monitoring remains maximized therefore other public users can able to access the same data that is identified by a particular individual. Hence in order to prevent the above mentioned condition it is essential to deviate the images and exact accuracy can be achieved with exact marked affected segments.

## 5 Conclusion

This article presents a method for detecting harmful substances in gastrointestinal processes that has proven to be effective. The proposed approach is based on an analytical model that incorporates differentiable variables through gradient functions. This analytical model is subsequently combined with a neural network to perform adjacent operation functions, resulting in a long-term monitoring system. The aforementioned integrated functions serve to mitigate loss in two distinct stages, namely, the training and testing phases. Furthermore, the information utilized for processing is sourced from diverse cordiality environments and a segmentation process is implemented. The proposed methodology is universally applicable for detecting any form of digestive system utilizing fundamental building blocks at a cost-effective rate. An essential feature of the projected method is the absence of any filtering methodology, as the utilization of segmented images results in superior error mitigation. However, alternative methods use a particular filter that impacts the capacity to collect information from specific images. Furthermore, the neural network exhibits a significantly higher processing speed when subjected to a reduced quantity of erroneous labels within the overall system. Various cases are employed to explicate the functionalities of the proposed approach in relation to the minimised loss function with summation terms. Conducting real-time testing through the utilisation of fundamental patterns yields favorable outcomes. Furthermore, the processing system can accommodate a substantial dataset in the future, thereby generating a sophisticated model that accurately identifies the entirety of digestive systems.

## Data Availability

The raw data supporting the conclusion of this article will be made available by the authors, without undue reservation.
